# Optimization of Celery Tail Waste-Based Hydrogels and Application in Soil Water Retention

**DOI:** 10.3390/gels11040248

**Published:** 2025-03-27

**Authors:** Yuqin Wang, Yuan Zhong, Jun Wu, Yufan Xie, Shiwei Fang, Liqun Cai

**Affiliations:** 1 College of Resources and Environmental Sciences, Gansu Agricultural University, Lanzhou 730070, China; yuqinrange1219@163.com (Y.W.); 18809453467@163.com (Y.X.); 13892801256@163.com (S.F.); cailq@gsau.edu.cn (L.C.); 2 State Key Laboratory of Aridland Crop Science, Gansu Agricultural University, Lanzhou 730070, China; zhongy@gsau.edu.cn

**Keywords:** celery tail waste, hydrogel, water absorption capacity, ratio optimization, soil water retention

## Abstract

In order to meet the demand for coordinated development of agricultural waste utilization and water-saving agriculture, this study utilized waste celery tailings (CT) to make a super-absorbent hydrogel by chemical cross-linking. The hydrogel was optimized and screened. The study demonstrated the optimal CT-gel synthesis method: 7.5 wt% CT, 0.05 wt% MBA cross-linker, and 70 °C for 2 h. The optimized gel had a water absorption of 708 g/g and a water retention of more than 20% at 25 °C on day 10. The soil water retention of the CT-gel increased with time and dosage. In sandy soils, 0.6% CT-gel was most effective. The pot experiment showed that 2% of the gel significantly increased the height and growth rate of radish seedlings. This study effectively utilized various components of CT and provided a scalable approach for converting agricultural waste into functional materials, which is valuable for arid soil improvement and sustainable agriculture.

## 1. Introduction

With the continuous increase in the global population and the rapid advancement of urbanization, the agricultural production sector is facing unprecedented severe challenges [1]. According to statistics from the Food and Agriculture Organization (FAO) of the United Nations, approximately 1.3 billion tons of agricultural waste worldwide are not effectively utilized each year, with vegetable tail waste accounting for more than 30% [2,3]. This phenomenon is particularly prominent against the backdrop of tight land resources and water shortages. The amount of tail waste generated during the annual vegetable production process is as high as 86 million tons [4]. If it is disposed of by traditional landfill methods, it would require an occupied land area of approximately 13,000 hectares (equivalent to the daily processing capacity of 18 standard football fields), and simultaneously release 1.2 million tons of carbon dioxide equivalents of greenhouse gases such as methane [5]. What is more serious is that the open-air stacking of tail waste can reduce the soil pH value by 0.8–1.2 units, leading to an imbalance in the microbial community [6]. At the same time, the areas where tail waste accumulates are prone to breeding mosquitoes, insects, and pathogens, becoming the source of pest and disease transmission and further threatening the safety of agricultural production. This current situation is in sharp contrast to the United Nations Sustainable Development Goal (SDG 12: Responsible Consumption and Production), prompting the academic community to actively explore new paths for the resource utilization of tail waste [7]. Therefore, exploring an efficient and environmentally friendly method for treating tail waste to achieve its reduction, harmlessness, and resource utilization is of great significance for the sustainable development of agriculture [8].

Celery (*Apium graveolens var. dulce*), a common edible vegetable, contains large amounts of cellulose (32–38%), lignin (10–14%), and pectin (7–8%), which can provide grafting active sites for the polyhydroxy skeleton of the hydrogel, as well as improve its mechanical strength and promote its swelling behavior. It is a good raw material for the preparation of high-performance hydrogels [9]. Celery tail waste, a common by-product of agricultural production, represents a significant waste of resources. For a long time, celery tail waste has not been fully utilized. Celery tail waste, rich in biomass components such as cellulose, hemicellulose, and pectin, as well as vitamins and minerals, provides a sustainable feedstock for hydrogel synthesis. These polysaccharides, though indigestible to humans, are valorized as functional matrices to enhance hydrogel performance [10]. Research by Wei et al. [11] shows that for every 1 ton of commercial celery produced, 0.35 tons of tail waste are generated. However, currently, less than 5% of the tail waste is used for feed or composting, and the vast majority is still disposed of by landfill, causing an economic loss of approximately 1.5 billion yuan each year [12]. Therefore, with the continuous enhancement of people’s awareness of environmental protection and resource recycling, how to turn celery tail waste into treasure and achieve its efficient utilization has become a focus of common concern in the scientific research and agricultural production fields. Pablo et al. [13] successfully converted broccoli tail waste into supercapacitor electrode materials, but there is still a gap in the research on the development of hydrogels from celery tail waste as a raw material.

Hydrogel is a polymer material with a three-dimensional network structure, and traditional hydrogels have great potential for application in many fields such as agricultural irrigation, horticultural moisturization, and soil improvement due to their ability to absorb and store large amounts of water [14]. In agricultural irrigation, hydrogels can slowly release stored water, reducing the number of irrigations and improving the efficiency of water utilization [15]. In horticultural moisturization, hydrogels can provide a stable water supply for flowers and seedlings to ensure healthy plant growth. It can be seen that the traditional hydrogel has demonstrated a strong application in various fields of agriculture due to its good swelling ability. However, most of its preparation methods rely on petrochemical raw materials, which not only make the production cost high but also exacerbate the consumption of non-renewable resources, contradicting the concept of sustainable development [16]. Harighi et al. [17] showed that traditional petroleum-based hydrogels, despite their high water absorption capacity, require energy-intensive production processes, limiting their sustainability in large-scale applications. In recent years, with the popularization of the concepts of green chemistry and sustainable development, the preparation of hydrogels using biomass resources has gradually become a research hotspot [18]. Compared with traditional chemical hydrogels, biomass hydrogels can retain the excellent properties of traditional hydrogels or even exceed them while utilizing green and renewable resources as raw materials. A bagasse hydrogel with a saturated swelling rate of 650 g/g (in ultrapure water) was constructed by building a 10–50 μm three-dimensional pore network, as studied by Gosukonda et al. [19]. It also increased the field water-holding capacity (the more stable soil water content that can be maintained by the soil profile) of potted pepper soil by 40% at −33 kPa substrate potential. Zhang et al. [20] found that a corn stover composite hydrogel can increase crop yield under drought conditions by 28%. In addition, traditional hydrogel preparation processes generally require high-temperature reaction environments and high doses of macromolecular agents, which greatly increase production costs and violate the principles of green chemistry. Biomass hydrogels can minimize the use of chemicals, get rid of the dependence on high-temperature environments, and reduce the process cost and environmental pollution. Therefore, the use of biomass to prepare green high-performance hydrogels is an effective way to reduce chemical pollution, reduce production costs, and follow the green chemistry of hydrogel preparation. This aligns with the future development trend of hydrogel preparation: cost reduction, efficiency improvement, and pollution reduction [21].

The aim of this study was to effectively utilize the agricultural waste celery tailings to improve the properties of the hydrogel, to provide experimental support for the development of environmentally friendly materials, and to apply them to soil [22]. The optimal ratio was determined by optimizing the raw material feeding ratio, comparative experiments, and data analysis methods [23]. On this basis, the effects of chemical reagent ratios on the water absorption properties of hydrogels were studied and analyzed, and the microstructure, swelling, and water retention properties of hydrogels were comprehensively evaluated. Finally, we chose radish seedlings as the study object and measured growth indexes, such as plant height and growth rate, during the growth process to evaluate the improvement effect of CT-gel on soil water retention. This experimental design not only helps to validate the potential application of hydrogels in agriculture but also provides a solid empirical basis for the application of hydrogels in wider fields such as environmental engineering [24].

## 2. Results and Discussion

### 2.1. Synthesis of the Hydrogel

**Figure 1:**
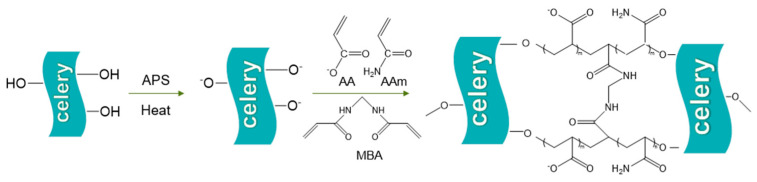
Synthesis process of CT-gel.

As shown in Figure 1, for the synthesis of hydrogels, ammonium persulfate (APS) was added as an initiator to the celery tail suspension to graft it onto the celery tail cellulose backbone. Ammonium persulfate (APS) was used as an initiator and added to the CT suspension to enable grafting onto the cellulose backbone of CT. Next, acrylic acid (AA) and acrylamide (AAm) monomers were introduced into the system and the copolymerization process was initiated under a nitrogen-protected atmosphere. In this process, the monomers were polymerized via a free radical polymerization mechanism. Subsequently, N,N′-methylenebisacrylamide (MBA) was introduced as a cross-linking agent, and a stable three-dimensional network structure was successfully constructed by cross-linking reaction driven by free radical polymerization. After the above synthesis steps, five hydrogels with different properties (Gel-1, Gel-2, Gel-3, Gel-4, Gel-5) and one hydrogel without the addition of celery taille (CK) were successfully prepared.

### 2.2. Analysis of Infrared Characteristics

**Figure 2:**
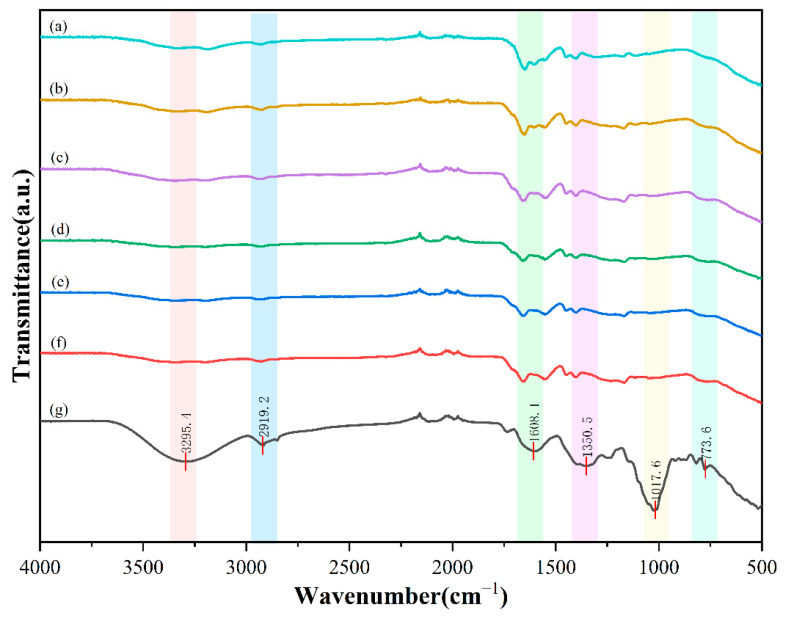
Fourier transform infrared spectroscopy characterization of (**a**) Gel-1, (**b**) Gel-2, (**c**) Gel-3, (**d**) Gel-4, (**e**) Gel-5, (**f**) CK, and (**g**) CT.

As shown in Figure 2, the successful construction of CT-gel (a–c) is attributed to the multi-scale structural synergy between natural fibers and synthetic polymers. The O-H stretching at 3350 cm^−1^, the C-O-C glycosidic bond at 1050 cm^−1^, and the C = O of the pectin ester bond at 1730 cm^−1^ in CT are partially retained in the modified hydrogel. However, the O-H peak expands to 2500–3300 cm^−1^, and there is an overlap between the peak at 1730 cm^−1^ and the C = O peak (1700 cm^−1^) of the carboxylic acid in PAA(Poly(acrylic acid)), indicating that the hydroxyl groups of cellulose and the carboxylic acid in PAA form an interpenetrating network through hydrogen bonds. At the same time, the oxidized cellulose may introduce additional carboxyl groups to enhance the interfacial compatibility [25]. Compared with CK, the changes in the peak shapes and the decrease in intensity of the peaks at 1650 cm^−1^ (amide I band of PAAm (Poly(acrylamide))) and 1550 cm^−1^ (amide II band) in samples a-e suggest that the MBA crosslinking agent reduces the free amide groups through covalent bonding, and the weakening of the characteristic peak of cellulose at 1050 cm^−1^ reflects that the cellulose segments are wrapped by the polymer [26]. In addition, the differential distribution of the peak intensity ratio between 1700 cm^−1^ (PAA) and 1650 cm^−1^ (PAAm) in samples a-e indicates that the (relative reactivity ratios) of AA and AAm monomers during the grafting process are regulated by the chemical microenvironment on the surface of the celery tail waste fibers, resulting in the heterogeneity of the spatial distribution of hydrophilic groups in different samples. The superposition and displacement of the characteristic absorption peaks in this natural-synthetic hybrid system confirm that CT not only acts as a physical filling phase but also participates in the construction of the three-dimensional network through chemical interactions, providing a molecular-level explanation for the enhanced water retention performance of the hydrogel [27].

### 2.3. Analysis of Microscopic Morphological Characteristics

The surface characteristics of CT, CT-gel, and CK were carefully examined using scanning electron microscopy (SEM). It was observed that CT, CK, and CT-gel all presented different morphological features.

**Figure 3:**
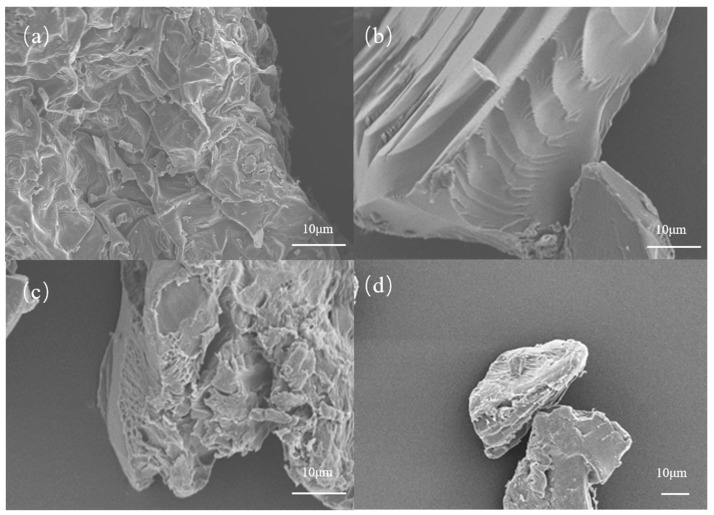
Surface characteristics of (**a**) CT, (**b**) CK, and (**c**,**d**) CT-gel.

As shown in Figure 3, the surface of the unmodified CT presented the typical characteristics of the natural plant matrix, characterized by sparse gully-like stomata and relatively smooth textures, reflecting its original physical state without chemical treatment. In contrast, due to the lack of structural inducing factors, CK showed a highly uniform and pore-free smooth surface, indicating that the uniformity of its crosslinked network led to the simplification of the microscopic topological structure. In comparison, CT-gel exhibited a significantly reconstructed multi-level pore network, with a dense distribution of interconnected pore structures of various shapes on its surface [28]. This implies that interfacial interactions (such as hydrogen bond crosslinking or physical entanglement) occurred between the plant fibers and the polymer matrix during the modification process, thus forming a heterogeneous composite three-dimensional porous framework. The generation of these multi-scale pores may be attributed to the template effect of the plant fibers during the gelation process [29]. The rough surface and the complex pore system of the plant fibers together increase the specific surface area of the material, providing abundant active sites and diffusion channels for the adsorption and storage of water molecules, which significantly optimizes the swelling behavior and water retention performance [30]. The phenomenon of the reconstruction of the microscopic structure indicates that the introduction of the natural plant matrix not only changes the chemical composition of the hydrogel but, more importantly, regulates its physical topological characteristics through the interfacial synergistic effect, providing theoretical support for the development of bio-based hydrogel materials with both environmental compatibility and high-efficiency functionality.

### 2.4. Analysis of the Water Absorption Rate of the Hydrogel

#### 2.4.1. Hydrogels with Different Addition Amounts of Celery Tail Waste

**Figure 4:**
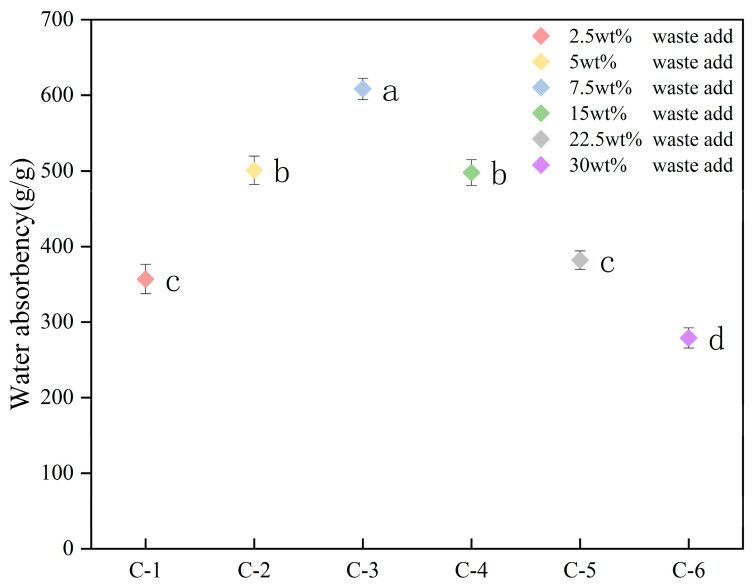
Nonlinear effect of the addition amount of CT on the equilibrium swelling ratio of CT-gel.

In this study, by systematically investigating the influence of different feed ratios on the water absorption performance of the hydrogel, significant regular characteristics were revealed. As shown in Figure 4, the water absorption rate of the hydrogel shows a non-linear relationship, first increasing and then decreasing with the change in the CT addition amount. The overall trend indicates that there is a clear optimization interval for the water absorption performance of the material. When the addition amount is 7.5%, the hydrogel reaches the performance peak, and the water absorption rate exceeds 600 g/g. This combination shows the best swelling ability, and it is speculated that it is related to the optimal crosslinking density of the three-dimensional network structure formed at this time. Further analysis shows that when the feed ratio is 5% and 15%, the water absorption rate is stable at the level of 500 g/g, showing a suboptimal water absorption characteristic. When the feed ratio is reduced to 2.5% or increased to 22.5%, the water absorption rate decreases to 350–370 g/g and 380 g/g, respectively, and the material performance is significantly weakened. Under the extreme feed ratio of 30%, the water absorption rate drops sharply to 270–290 g/g, reflecting the destructive effect of excessive monomers on the polymer network structure. It can be seen that too low an addition amount of waste will lead to insufficient effect as a crosslinking skeleton, resulting in a loose network structure that cannot effectively lock water. Conversely, too high an addition amount of waste will cause the waste particles to block the pores or form a non-uniform structure, reducing the effective adsorption area. When the addition amount of waste is moderate, the active groups (such as hydroxyl groups, carboxyl groups, etc.) contained in it form a moderate three-dimensional network structure with the crosslinking agent. This structure can not only combine with water molecules through hydrophilic groups but also has sufficient elasticity to accommodate a large amount of water. An even pore structure can be formed through graft copolymerization at the best ratio, making the water absorption rate reach the peak.

Therefore, when the feed ratio is 7.5%, the water absorption rate reaches its peak. The synergistic effect between the moderate crosslinking density of the three-dimensional network structure and the hydrophilic groups can significantly enhance the swelling ability. However, an excessive feed ratio will lead to pore blockage and uneven crosslinking, and the water absorption rate drops sharply to 270–290 g/g, which confirms the conclusion of Hou et al. [31] that an excessive amount of biomass can trigger network collapse. Compared with the unmodified control group (CK group, 380 g/g), the water absorption rate of the group with a 7.5% feed ratio increases by 58%, and the statistical difference is significant (*p* < 0.01), further verifying the feasibility of optimizing the performance of hydrogels by utilizing the whole components of agricultural waste [32]. Although the 7.5% concentration showed the highest overall performance, finer concentration gradients may further optimize the extreme points, which will be the focus of subsequent studies.

#### 2.4.2. Hydrogels with Different Proportions

**Figure 5:**
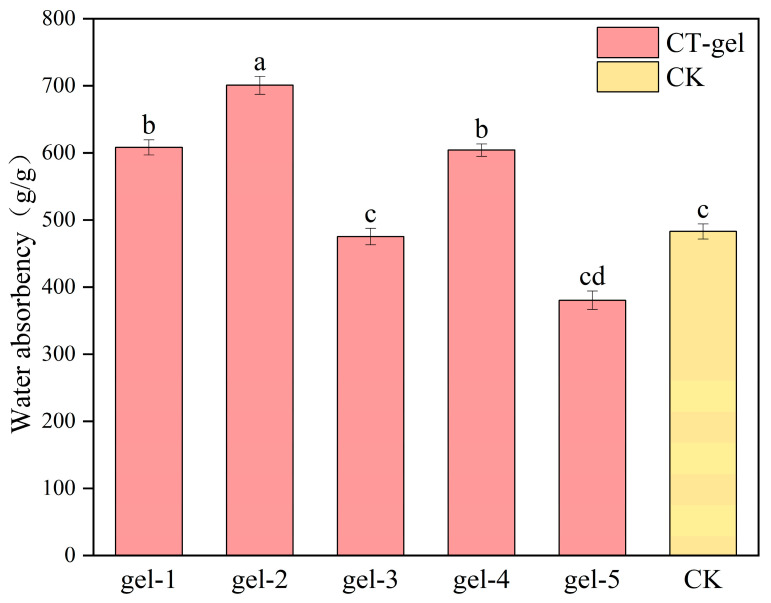
Water absorption multiples of CT-gel under different treatments. Different letters in the figure represent different significance level groupings. The same letter indicates that there is no significant difference at this level, while different letters indicate significant differences. Starting from the letter “a” in alphabetical order, the further the letter is in the alphabet, the relatively greater the degree of difference it represents.

As shown in Figure 5, the water absorption performance of the hydrogels exhibits a significant component-dependent characteristic, and its modification effect is complexly influenced by material ratios and structural regulation. Compared with the CK group without the addition of celery waste, the Gel-1, Gel-2, and Gel-4 samples show high water absorption rates. In particular, Gel-2 has an even higher water absorption rate, indicating that the introduction of CT optimizes the network porosity and the distribution of hydrophilic groups through physical-chemical synergistic effects. However, the performance degradation of Gel-3 and Gel-5, especially that Gel-5 is significantly lower than the CK group, implies that excessive or specific ratios of plant fibers may disrupt the uniformity of the cross-linked network, resulting in a dense structure that inhibits the swelling kinetics. Statistical analysis further reveals that the water absorption capacity of Gel-2 is significantly different from that of other treatment groups, reflecting the matching of its unique spatial arrangement of hydrophilic groups with pore connectivity. The sharp decline in the water absorption capacity of Gel-5 implies the existence of a critical ratio threshold in the natural-synthetic hybrid system. The research results indicate that by precisely regulating the ratio of CT to synthetic monomers and the cross-linking density, hydrogel materials with both high water absorption and structural stability can be directionally constructed. This result is similar to the research of Wojciech et al., verifying the feasibility of the full-component utilization of agricultural waste [33].

### 2.5. Analysis of Swelling Capacity of Hydrogels at Different pH

**Figure 6:**
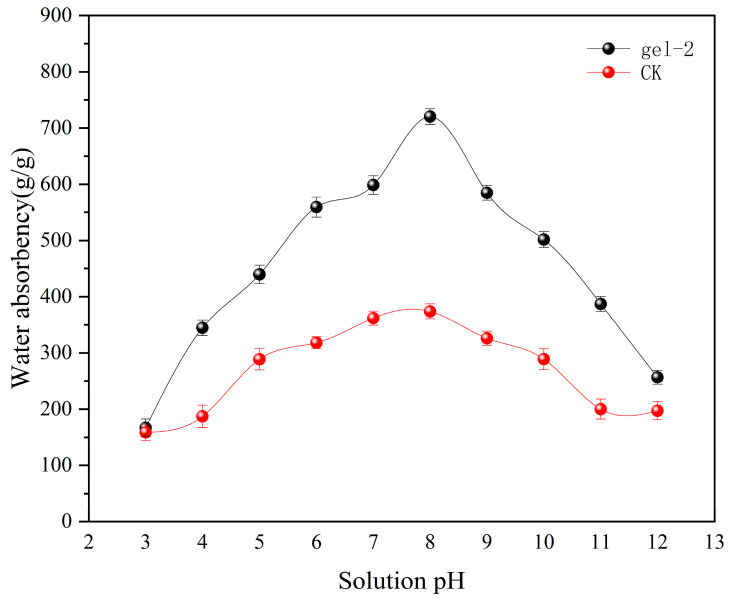
Water absorption curves of CT-gel and CK under different pH values.

The experimental results are shown in Figure 6, the water absorption properties of hydroxyl gels (Gel-2 and CK) both exhibit pH-dependent characteristics, but there is a significant difference in the response mechanisms of the two. The water absorption multiple of Gel-2 shows an obvious unimodal curve with the change of the pH value, reaching a peak value of 708 g/g under weakly alkaline conditions (pH ≈ 8), which is nearly double that of CK (the peak value is about 350 g/g at pH 7–8). It still maintains a relatively high water absorption capacity in the acidic (pH < 7) and strongly alkaline (pH > 10) ranges (for example, when pH = 5, the water absorption multiple of Gel-2 is 454 g/g, while that of CK is only 273 g/g). Especially in the neutral to weakly alkaline range (pH 7–10), the water absorption advantage of Gel-2 is the most significant (for example, when pH = 9, Gel-2 still maintains 600 g/g, while CK has dropped to 300 g/g), indicating that the ionized groups (such as carboxylic acid or sulfonic acid groups) in its molecular chains fully expand the network structure through electrostatic repulsion under specific pH conditions, enhancing the swelling ability. In contrast, the water absorption performance of CK is less sensitive to pH changes, and the curve is relatively gentle, which may be related to its higher crosslinking density or differences in the types of functional groups. This result reveals the environmental adaptability advantage of Gel-2 in a wide pH range, providing an important basis for its application in complex acid-base environments (such as contaminated soil remediation or drug-controlled release), but its structural stability under extreme pH conditions still needs to be further verified.

### 2.6. Analysis of Water Retention and Reusability Characteristics

**Figure 7:**
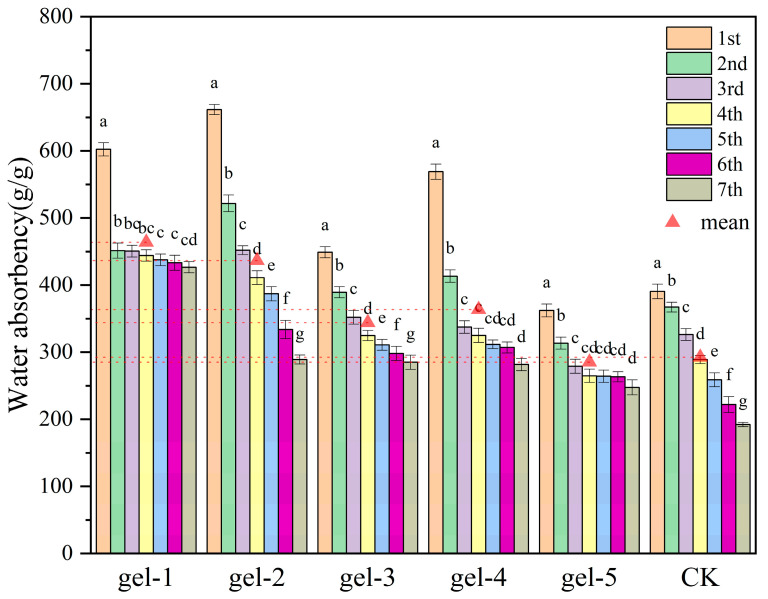
Reusability characteristics of various hydrogels under different treatment conditions at room temperature. In the bar graphs, the letters (a–g) indicate the level of significant difference between the groups represented by each bar, where “a” indicates the group with the highest level of significant difference, followed by “b”, “c” and “d” until “g”, which denotes the groups with sequentially lower levels of significant difference.

The experiments on hydrogel reusability are shown in Figure 7, where different hydrogels show significant performance differences during multiple water absorption. Among them, Gel-1 has the best comprehensive reusability, with the highest average water absorption rate (about 600 g/g). The decrease in dissolution rate was smaller each time when water was absorbed multiple times, indicating a strong stability and structural durability of the water absorption properties. Although the initial water absorption rate of Gel-2 exceeds 700 g/g, there are large subsequent fluctuations and obvious performance degradation, which may be related to the damage to the material structure during cyclic use. Gel-3 and Gel-4 maintain relatively high levels of 400–500 g/g and close to 600 g/g, respectively, during multiple uses, but Gel-4 shows a slow downward trend. The repeated water absorption capabilities of Gel-5 (300–400 g/g) and the control group CK (significantly lower than the hydrogel groups) are weak. Overall, the multiple water absorption performance of the hydrogels shows a common pattern of “high initial effect and subsequent slow decline”. However, Gel-1 demonstrates better potential for cyclic use by balancing a high water absorption rate and structural stability, which is closely related to its crosslinked network design or component optimization, providing an important reference for the development of reusable water-absorbing materials [34].

**Figure 8:**
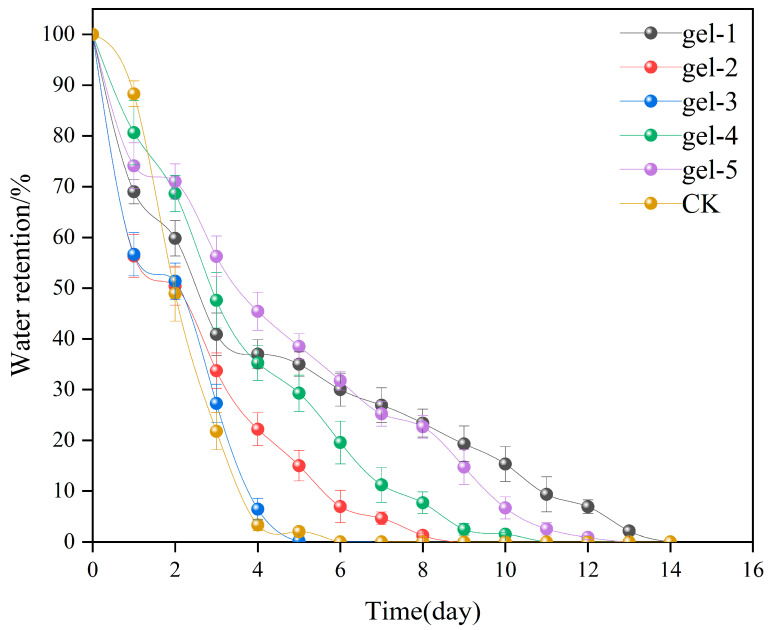
Water retention capacities of various hydrogels under different treatment conditions at room temperature.

The water retention of the hydrogels was analyzed as shown in Figure 8, and the water retention of the CT-gel (Gel-1 to Gel-5) was significantly better than that of the control. In the initial stage (Day 0), the water retention rates of all samples are 100%, and the water contents are uniform. As time goes by, the water retention rate of the control group drops sharply, while the hydrogel samples exhibit gradient water retention characteristics. Among them, Gel-1 has the best water retention performance. The water retention rate is still higher than 20% on the 10th day, indicating that its three-dimensional network structure can effectively lock water and inhibit evaporation. The water retention performance of Gel-3 is weak, and the water retention rate is already lower than 20% on the 6th day, which may be related to the increased water diffusion rate caused by insufficient crosslinking density or poor pore connectivity. The water retention rates of all samples show the kinetic characteristics of “rapid decline in the early stage and gradually leveling off in the later stage”. The water retention rate decreases by 50–70% in the early stage (0–3 days), and the decrease range narrows to 10–20% in the later stage (3–10 days). This phenomenon is attributed to the two-stage mechanism of the rapid evaporation of free water on the surface of the hydrogel and the slow release of bound water inside. Overall, the water retention capacity of the hydrogel is closely related to the stability of its crosslinked network, the closure of pores, and the retention rate of hydrophilic groups. The high-efficiency water retention characteristics of Gel-1 provide an experimental basis for its application in arid agriculture [35].

### 2.7. Analysis of the Water Retention and Water-Holding Capacities of Hydrogels in Different Soils

**Figure 9:**
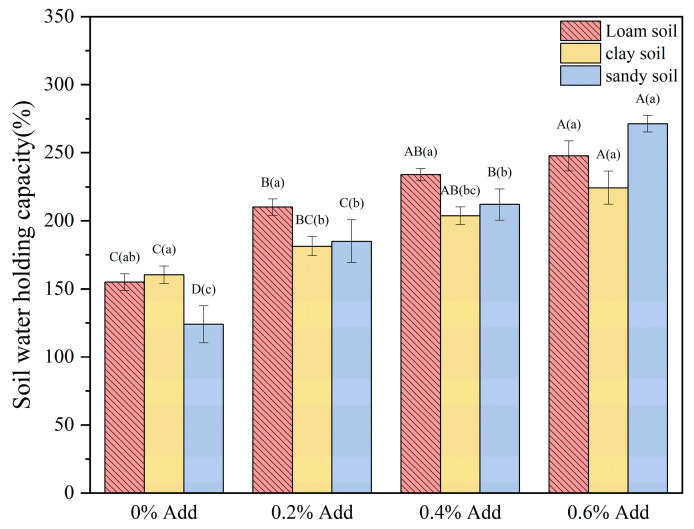
Water-holding capacities in different soils with different addition amounts of Gel-1. The letters (a–c, A–D) on the labels are the results of the significant difference analysis. Among them, the capital letters represent the analysis results of different addition amounts of the same type of soil, and the lowercase letters represent the analysis results of different types of soil with the same addition amount of hydrogel.

As shown in Figure 9, as the addition amount of the hydrogel increases from 0% to 0.6%, the water-holding capacities of loam soil, clay soil, and sandy soil all show a significant increasing trend, and the increase is the largest when the addition amount is 0.6%, verifying the positive correlation between the addition amount of the hydrogel and the water-holding capacity. Under the condition of the same addition amount, there are significant differences in the water-holding capacities of soils with different textures: due to the large pores in sandy soil, it may be more conducive for the hydrogel to enter the pores and form a better water retention network. Therefore, when the addition amount is 0.6%, the water-holding capacity is the highest, reaching 270%, followed by loam soil (244%). The dense structure of clay soil may limit the swelling space of the hydrogel, resulting in a relatively weak water-holding performance (221%). It is worth noting that even when no hydrogel is added, there are differences in the baseline water-holding capacities of the three types of soil. However, after the addition, the water-holding capacities of all soil types are effectively enhanced, proving that the hydrogel can improve the water retention capacity of soils with different textures through its high water absorption characteristics. This characteristic provides an important theoretical basis for optimizing soil management in arid regions and improving the efficiency of water resource utilization. However, in practical applications, it is necessary to carry out refined regulation in combination with the synergistic effects of the soil type and the addition amount [36].

**Figure 10:**
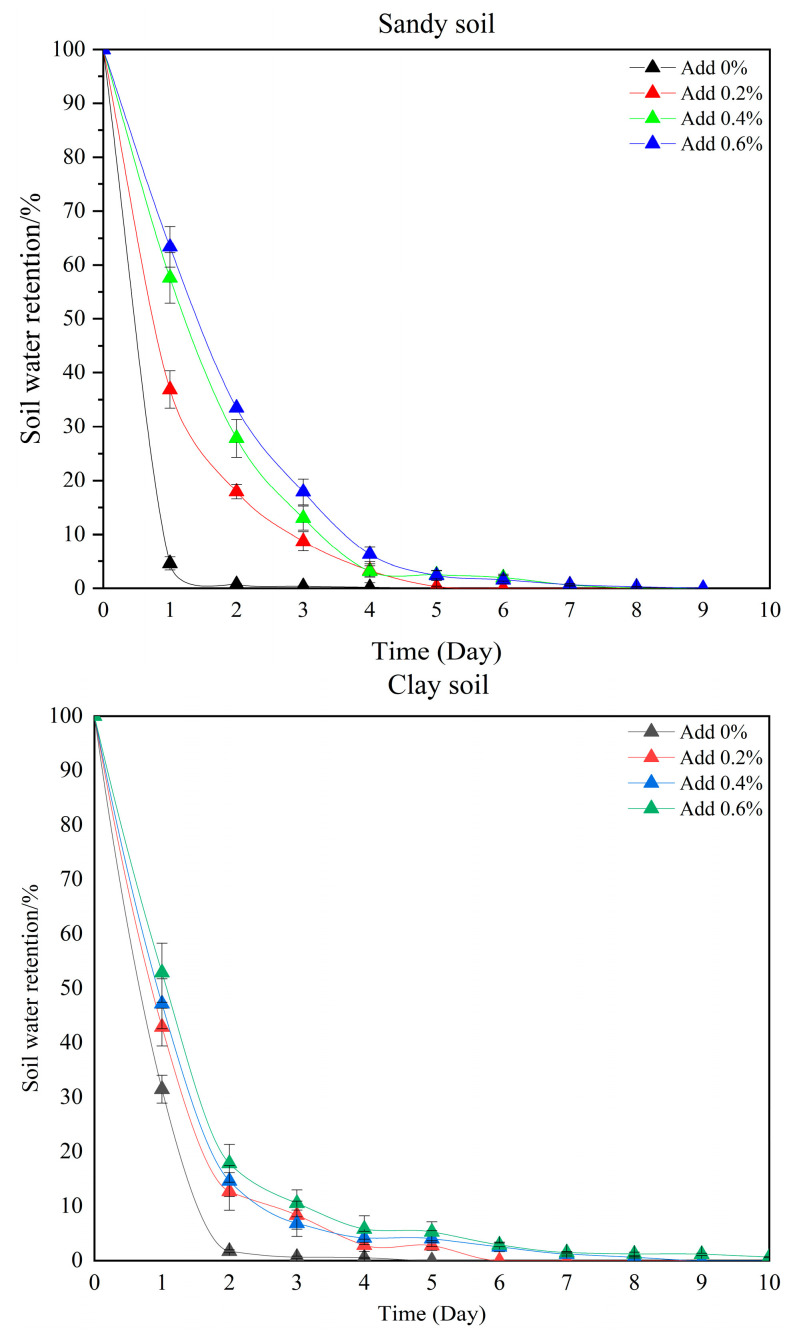

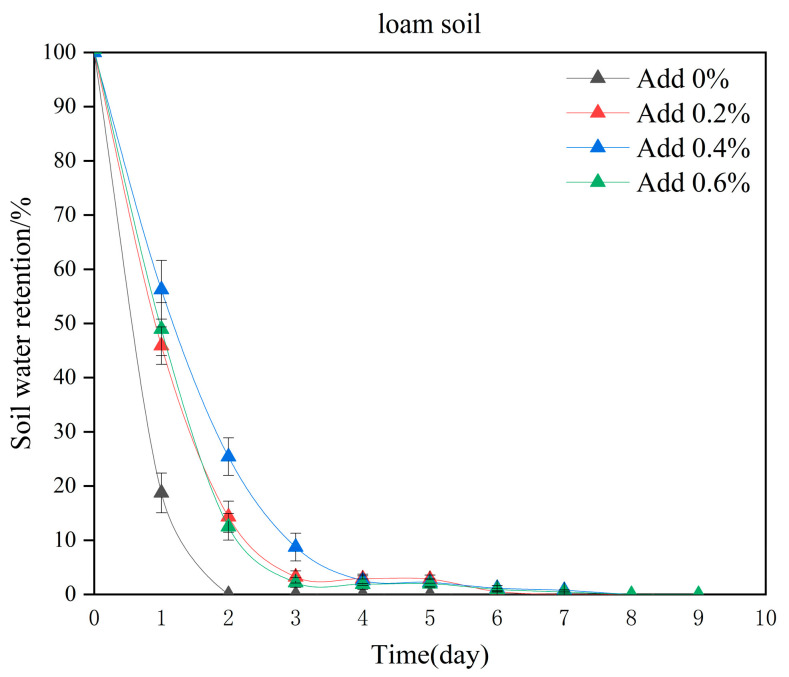
Water retention properties in different soils with different addition amounts of Gel-1.

The experimental results, as shown in Figure 10, indicate that the hydrogel had a significant time-dependent and additive gradient effect on the improvement of soil water retention properties. In loam soil, clay soil, and sandy soil, the addition of hydrogels can all delay the rate of soil water loss. Among them, the higher the addition amount (0.6% > 0.4% > 0.2%), the slower the decrease in the water retention rate, and the effect is enhanced over time. In the initial stage (0–3 days), the water retention rates of all soils decrease rapidly. However, the high addition amount group (such as 0.6%) can still maintain a relatively high water retention rate in the later stage (3–10 days), while the group without addition (0%) loses water sharply to below 20% or even approaches dryness in a short period (2–3 days for sandy soil and 3 days for clay soil). The type of soil has a significant impact on the effect of the hydrogel: due to its large porosity, sandy soil has the fastest water loss rate, but 0.6% of the hydrogel can still maintain its water retention rate at 10% on the fifth day. Although clay soil has relatively high initial water retention due to its dense particles, the swelling space of the hydrogel is limited, and the water retention effect under a high addition amount is weaker than that in loam soil. Loam soil, because it has both sandy and clay characteristics, forms the optimal water retention network, and its water retention persistence is the most prominent when the addition amount is 0.6%. It is worth noting that the hydrogel shows a two-stage characteristic of “delaying water loss in the early stage and maintaining a low-level stability in the later stage” in all types of soil. Its water retention mechanism may be related to the synergistic effect of the water absorption–water release kinetics of the hydrogel and the pore size distribution of the soil. This discovery provides a scientific basis for precisely regulating the addition strategy of hydrogels (for example, sandy soil in arid regions requires a higher addition amount), but the long-term water retention efficiency and environmental adaptability still need further research [37].

### 2.8. Evaluation of the Plant Growth Performance of Hydrogels

**Figure 11:**
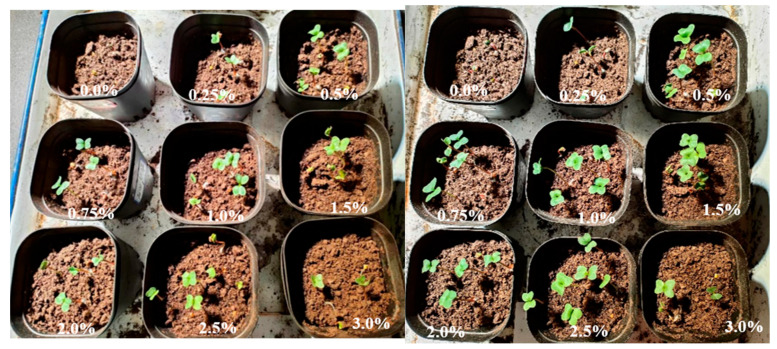
Growth of radish seedlings on day three (**left**) and day five (**right**).

**Figure 12:**
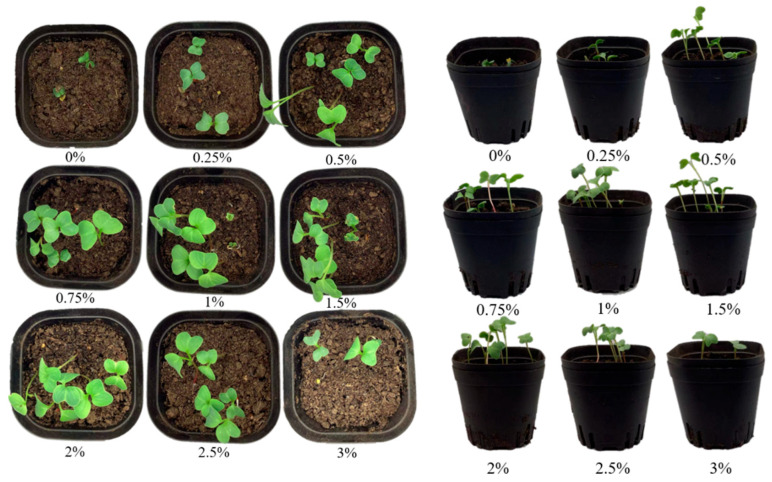
Number of leaves and growth height of radish seedlings on the seventh day.

The aim of the study was to investigate the effect of different concentrations (0%, 0.25%, 0.5%, 0.75%, 1%, 1.5%, 2%, 2.5% and 3%) of hydrogel on the growth of radish seedlings under drought stress conditions. The results are shown in Figure 11 and Figure 12. The experiment was carried out in a simulated drought environment, and the radish seedlings were planted and their growth conditions were carefully monitored, with the aim of evaluating the role of hydrogels in improving the growth conditions of the seedlings.

Under water-limited conditions, the appropriate addition of hydrogels can significantly improve the growth performance of radish seedlings, but the effect is obviously concentration-dependent. The experiment shows that when the addition amount of the hydrogel is 0.5–2.5%, the water retention capacity of the soil is the best. The germination rate of radish seedlings increases by 15–20%, the height of the seedlings increases by 3–5 cm, the main root extends by 2–3 cm, the number of lateral roots increases by about 30%, and the leaves grow more vigorously. This improvement is derived from the slow water release system formed by the hydrogel, which can not only maintain the stable humidity required for seed germination but also promote root respiration and nutrient absorption by optimizing the soil pore structure. However, excessive addition (such as 3%) will instead inhibit growth, possibly because the excessive water absorption and swelling of the gel lead to a decrease in soil air permeability, or it competes with plants for limited water resources. The seedlings in the optimal concentration group (2%) exhibit overall coordinated morphological development, confirming that the rational application of hydrogels can improve the growth of radish seedlings under drought stress. The research results provide a practical solution for water-saving cultivation in arid regions, but it is necessary to further optimize the application standards in combination with the specific soil texture [38].

## 3. Conclusions

In this study, we investigated the water absorption and water retention properties of hydrogels, as well as their effects on the growth of radish seedlings. The results showed that the performance of hydrogel was significantly affected by the composition, pH value, time, and amount of addition and followed a clear pattern of change.

Among the many formulations, Gel-2 with a 7.5% addition of raw materials showed excellent water absorption performance, with the water absorption rate exceeding 600 g/g. Especially in the weakly alkaline environment with a pH value of about 8, the water absorption multiplication rate was as high as 708 g/g, showing good environmental adaptability. However, the performance of Gel-2 is not stable after repeated use, and its performance decreases after repeated use. Although the initial water retention was slightly lower than that of Gel-1, it had the best overall reuse performance, maintaining an average water retention of about 600 g/g, with little fluctuation over the seven applications, and significantly higher water retention than the other treatments, with water retention still higher than 20% on the 10th day. In soil application, the water-holding capacity of Gel-1 was up to 270% at 0.6% in sandy soil. In the experiment of promoting the growth of radish seedlings under drought stress, the effect of adding 0.75–2% was significant, with the 2% concentration group showing the most harmonized morphological development of the seedlings. This finding provides a feasible solution for CT utilization, crop cultivation in arid regions, and water management.

## 4. Materials and Methods

### 4.1. Materials

The raw material used was celery (*Apium graveolens* L.) tail waste, sourced from the cafeteria of Gansu Agricultural University.

The selected reagents were as follows: acrylic acid (AA), acrylamide (AAm), ammonium persulfate (APS), N, N′-methylene bisacrylamide (MBA), and sodium hydroxide (NaOH).

The sandy, loamy, and clay soils tested were sampled at a depth of 0–20 cm. Three samples of each type were taken, each weighing 150 g and dried naturally, and then sieved through a 0.25 mm sieve to test the water-holding and water-retaining properties of the different ratios. The soil used for growing radish seedlings was potting soil (purchased horticultural nutrient soil).

### 4.2. Preparation of Hydrogels

The tail waste was added to water, and tail waste suspensions with different feeding ratios were prepared using a juicer and homogenized with a digital disperser (LC-OES-200SH, manufactured by Hunan Lichen Technology Co., Ltd., Changsha, China) [39]. A total of 384 g of the tail waste suspension was added to a 1-L three-necked flask equipped with a mechanical stirrer and a nitrogen pipeline. The tail waste suspension was purged with nitrogen for 30 min and then heated to 70 °C under a nitrogen stream. The initiator APS was added, and the temperature was maintained at 70 °C. After 30 min, aqueous solutions of a predetermined amount of AAm, AA (neutralisation with 40% aqueous NaOH solution), and the cross-linking agent MBA were added. Six hydrogel products (C-1, C-2, C-3, C-4, C-5, and C-6) were synthesized by changing the addition amount of different raw materials (2.5wt%,5 wt%, 7.5 wt%, 15 wt%, 22.5 wt%, and 30 wt%). By measuring the water absorption of hydrogels with six different raw material addition amounts, the hydrogel with the best water-absorbing performance was selected to adjust the ratio between monomers, initiators, and cross-linkers. Subsequently, five hydrogels with different ratios (Gel-1, Gel-2, Gel-3, Gel-4, Gel-5) and CK were synthesized for subsequent performance testing. The details of the casts are shown in Table 1.

The reaction was carried out under a nitrogen-filled environment and heated for 3 h. The product obtained was dried in a drying oven at 45 °C and ground into powdered granules to be used as material for subsequent experiments.

### 4.3. Determination of the Swelling Ratio of Hydrogel

The swelling capacity of the hydrogels was determined by the tea bag method. A total of 0.05 g of powder was placed in a pre-weighed and moistened tea bag, and then the hydrogel in the tea bag was immersed in ultrapure water at room temperature for a certain period of time to reach the equilibrium of swelling. Finally, the tea bags were removed and suspended until no water droplets fell, and the excess liquid was filtered off with filter paper to measure the equilibrium swelling rate (Q_eq_) [40].

Meanwhile, in order to study the effect of different pH on the swelling capacity, 0.1 g of hydrogel was weighed into a pre-weighed wet tea bag and immersed into solutions with pH values from 3 to 12, respectively, to determine the Q_eq_ at each pH value and to plot Q_eq_ as a function of pH [41].

(1) $$Q_{eq}=\frac{W_{eq}-W_{0}}{W_{0}}$$

where Q_eq_ is the water absorption, W_eq_ is the weight of the swollen sample after reaching equilibrium, and W_0_ is the weight of the dried sample.

### 4.4. Infrared Spectroscopy

Using a Fourier transform infrared spectroscopy analyzer (FTIR-650 model, manufactured by Tianjin Gangdong Technology Development Co., Ltd., Tianjin, China), the powders of CT, CT-gel, and CK were rapidly pressurized under a pressure of 10–15 MPa, and the infrared spectra of the samples were measured in the wavelength range of 400–4000 cm^−1^.

### 4.5. SEM

A small amount of the dried CT and hydrogel samples were taken, and the contrast agent was gold plating. The surface morphology of the samples was observed using a scanning electron microscope (JEOL S-3400N model, manufactured by HITACHI in Tokyo, Japan) at an accelerating voltage of 5 kV [42].

### 4.6. Determination of the Reusability of the Hydrogel

A total of 0.1 g of the hydrogel was placed in a pre-weighed wetted tea bag, and the initial mass was carefully recorded. Then, the sample was immersed in ultrapure water and left standing for 24 h to reach the swelling equilibrium, and the mass m_1_ was measured to obtain the swelling ratio [43]. Then, the hydrogel sample that had reached the swelling equilibrium was transferred to an oven, and the temperature was maintained at 75 °C for 6 h [44]. After ensuring that the hydrogel was completely dehydrated, it was allowed to absorb water again to reach the swelling equilibrium. To ensure the accuracy and repeatability of the data, this comprehensive test scheme was continuously repeated seven times [45].

### 4.7. Determination of the Water Retention and Water-Holding Capacity of the Hydrogel

The hydrogel that had reached the swelling equilibrium was placed in a room temperature environment (25 °C, humidity 20%), and then the weight of the hydrogel was recorded for 20 consecutive days to obtain the water retention curve of the hydrogel. Finally, the hydrogel with the best water retention performance was selected for subsequent experimental measurements to determine its characteristics in different soils [46]. The soils used in the experiment were sandy soil, loam soil, and clay soil. A total of 30 g of soil was taken and mixed with 0%, 0.2%, 0.4%, and 0.6% of the hydrogel powder (W_s_), respectively, and put into a flower pot (W_0_). Then, the experimental materials (the flower pot and the mixed sample) were immersed in ultrapure water for one day. After 24 h, the materials were taken out and weighed, which was recorded as W_1_. Subsequently, it was placed at room temperature and weighed every day to observe its changes, which was recorded as Wt. The dry weight after reaching a constant weight was recorded as W_dry_, and curves of the water-holding rate (W_h_) and water retention (W_r_) were plotted based on the data [47].

(2) $$W_{h}=\frac{W_{1}-W_{0}}{W_{s}}\times 100\%$$

(3) $$W_{r}=\frac{W_{t}-W_{dry}}{W_{1}-W_{dry}}\times 100\%$$

where W_h_ is soil water-holding capacity, and W_r_ is soil water retention.

### 4.8. Pot Experiment

30 g of potting soil was weighed, and 0%, 0.25%, 0.5%, 0.75%, 1%, 1.5%, 2%, 2.5%, and 3% of the hydrogel were added, respectively, for planting radish seedlings. The seeds of the radish seedlings were washed three times with clean water and soaked for 10 h [48]. After the seeds showed a white tip, they were sown into the potting soil added with the hydrogel and placed in an incubator at a temperature of 23 °C and a humidity of 40%. Before germination, the seeds needed to be sprayed with water every day to ensure that the seeds had sufficient moisture. After the seeds germinated, the spraying was stopped, and the seedlings were taken out of the incubator and placed indoors. The growth of the radish seedlings was observed and photographed for record [49]. The height of the radish seedlings when they stopped growing was measured.

## Figures and Tables

**Table 1:** Design of feed ratios for hydrogel synthesis with different initiator, crosslinker, and monomer ratios.

Number	AA (wt %)	AAm (wt %)	MBA (wt %)	APS (wt %)	Water (g)
CK	7	3	0.025	0.2	124.144
Gel-1	7	3	0.05	0.2	124.000
Gel-2	7	3	0.025	0.2	124.144
Gel-3	7	3	0.1	0.2	123.712
Gel-4	5	5	0.05	0.2	126.560
Gel-5	3	7	0.05	0.2	129.120

## Data Availability

The data supporting the findings of this study are available within the article and its tables/figures. No additional external datasets were generated or analyzed.
